# The Auditory Anatomy of the Minke Whale (*Balaenoptera acutorostrata*): A Potential Fatty Sound Reception Pathway in a Baleen Whale

**DOI:** 10.1002/ar.22459

**Published:** 2012-04-10

**Authors:** Maya Yamato, Darlene R Ketten, Julie Arruda, Scott Cramer, Kathleen Moore

**Affiliations:** 1 Woods Hole Oceanographic Institution Biology DepartmentWoods Hole, Massachusetts; 2 Harvard Medical School Department of Otology and LaryngologyBoston, Massachusetts; 3 Massachusetts Eye and Ear InfirmaryBoston, Massachusetts; 4 International Fund for Animal Welfare Marine Mammal Rescue and ResearchYarmouth Port, Massachusetts

**Keywords:** cetacea, mysticete, hearing, ear, acoustic fat, imaging

## Abstract

Cetaceans possess highly derived auditory systems adapted for underwater hearing. Odontoceti (toothed whales) are thought to receive sound through specialized fat bodies that contact the tympanoperiotic complex, the bones housing the middle and inner ears. However, sound reception pathways remain unknown in Mysticeti (baleen whales), which have very different cranial anatomies compared to odontocetes. Here, we report a potential fatty sound reception pathway in the minke whale (*Balaenoptera acutorostrata*), a mysticete of the balaenopterid family. The cephalic anatomy of seven minke whales was investigated using computerized tomography and magnetic resonance imaging, verified through dissections. Findings include a large, well-formed fat body lateral, dorsal, and posterior to the mandibular ramus and lateral to the tympanoperiotic complex. This fat body inserts into the tympanoperiotic complex at the lateral aperture between the tympanic and periotic bones and is in contact with the ossicles. There is also a second, smaller body of fat found within the tympanic bone, which contacts the ossicles as well. This is the first analysis of these fatty tissues' association with the auditory structures in a mysticete, providing anatomical evidence that fatty sound reception pathways may not be a unique feature of odontocete cetaceans. Anat Rec, 2012. © 2012 Wiley Periodicals, Inc.

The transition to aquatic life resulted in several modifications to the auditory anatomy of cetaceans. Cetaceans lack external pinnae, and the external auditory canal has been reduced to a very narrow channel. The middle and inner ear migrated laterally out from the skull and are encased in the dense tympanoperiotic complex (Hunter, 1787; Eschricht and Reinhardt, 1866; Kernan, 1919). Other characteristics of the auditory system are specific to each suborder. The gross auditory anatomy and hearing pathways in Odontoceti (toothed whales) have been relatively well described. In odontocetes, the external auditory canal is considered vestigial (Reysenbach De Haan, 1957; Dudok Van Heel, 1962; Norris, 1968; McCormick et al., 1970). Bone conduction is thought to play a minor role because there is no osseous connection between the tympanoperiotic complex and the rest of the skull in most odontocete species (Claudius, 1858, in Yamada, 1953; Ketten and Wartzok, 1990; Nummela et al., 2007). In addition, the air spaces around the tympanoperiotic complex are thought to provide acoustic insulation from the rest of the skull, which may be important for directional hearing (Reysenbach De Haan, 1957).

A more likely mechanism for sound reception in odontocetes is via perimandibular “acoustic” fat bodies that are in direct contact with the ears, including both the tympanic and periotic bones (Norris, 1964; Ketten, 1994, 1997; Ridgway, 1999; Cranford et al., 2010). Although odontocetes receive sounds across various locations on the head (Bullock et al., 1968; Brill, 1988; Mohl et al., 1999; Mooney et al., 2008; Cranford et al., 2008a), these biochemically distinct fats are thought to act as a preferential pathway of sound from the environment to the ears (Norris, 1964; Bullock et al., 1968; Varanasi and Malins, 1971; Litchfield et al., 1975; Brill et al., 1988; Koopman et al., 2006; Zahorodny et al., 2009).

These odontocete “acoustic fats” are composed of multiple lobes, including the inner lobe filling the enlarged mandibular hiatus and the outer lobe covering the lateral and ventral portions of the mandible (Norris, 1968; Ketten, 1994, 1997; Ridgway, 1999). In addition to these two fat lobes, which are located anterior to the tympanoperiotic complex, there is also increasing evidence for a third fat channel located lateral to the tympanoperiotic complex. In an electrophysiological study focused on striped dolphins (*Stenella coeruleoalba*), Bullock et al. (1968) found that the lateral area near the external auditory meatus opening was sensitive to low-frequency sounds below 3 kHz. Renaud and Popper (1975) also found that the region near the external auditory meatus opening was more sensitive to lower frequency sounds (below 20 kHz) in a behavioral study on bottlenose dolphins (*Tursiops truncatus*). Furthermore, Ketten (1994) provided anatomical evidence for a distinct lateral fat channel by applying magnetic resonance imaging (MRI) techniques to multiple odontocete species. Most recently, Popov et al. (2008) used auditory brainstem response latencies to advance the hypothesis that there are two acoustic windows in the bottlenose dolphin. The acoustic window was calculated to be near the external auditory meatus opening at frequencies below 22 kHz, while sounds above 32 kHz were received through the lower jaws.

The pathways of sound reception are unknown in Mysticeti (baleen whales), and there have been no reports of sound-conducting fats similar to those of odontocetes. The small opening to the external auditory meatus is visible on the surface, as in odontocetes. However, researchers disagree on whether the auditory canal is continuous from the opening of the external auditory meatus to the tympanic membrane and whether it is a functional part of the auditory system (Carte and Macalister, 1868; Yamada, 1953). At the end of the auditory canal is the “glove finger,” an everted, extended, thickened tympanic membrane, the function of which remains unclear (Lillie, 1910; Fraser and Purves, 1960). This elongated glove finger is not found in odontocetes or any other mammals. Another major difference between odontocete and mysticete ears is the connection of the tympanoperiotic complex with the skull. In mysticetes, the posterior flange of the periotic bone is wedged against the squamosal and the exoccipital bones (Yamada, 1948; Fig. 1). The anterior flange of the periotic is also firmly embedded in the squamosal bone, reducing the acoustic isolation of the tympanoperiotic complex. Bone conduction has not been dismissed as a potential sound reception pathway in baleen whales (Ketten, 1992, 2000).

**Fig 1:**
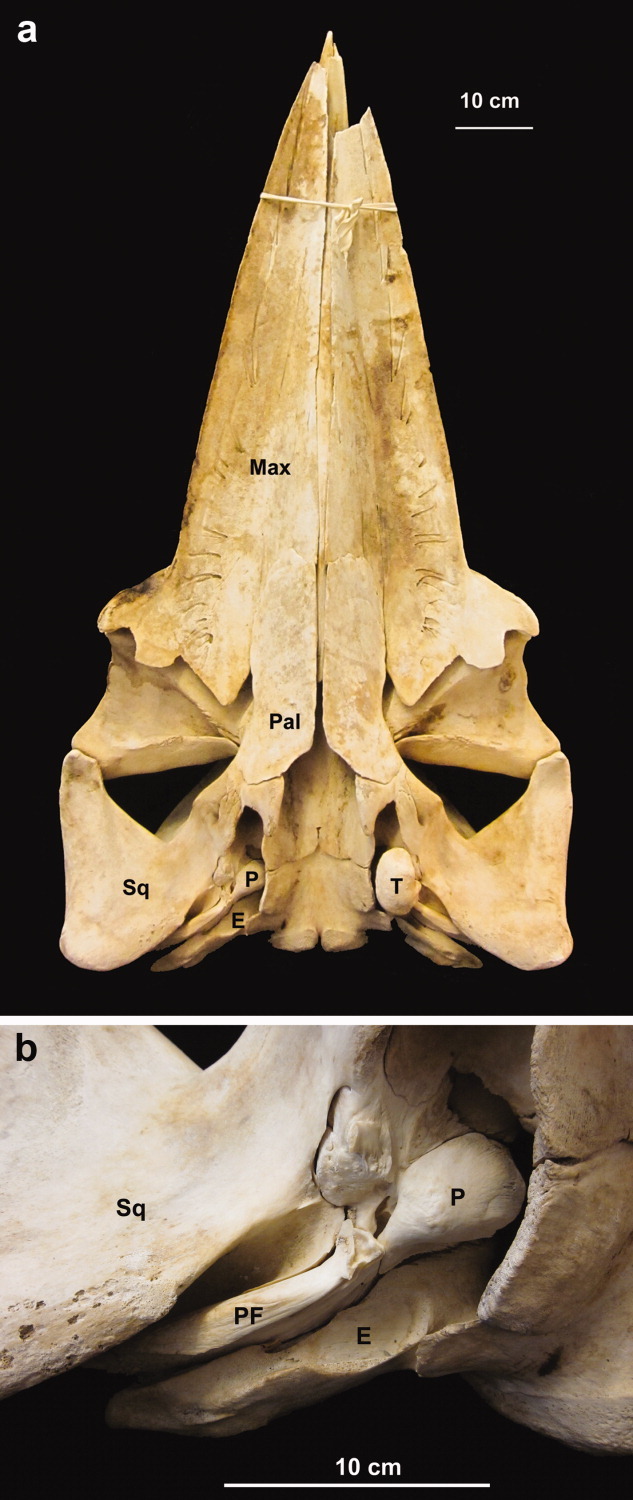
Photograph of a minke whale skull (B-acu21; not part of our study). (a) Ventral view of the skull, where the mandibles have been removed. The tympanic bone has been removed on the right side of the animal (left side of the photograph) to expose the periotic bone. (b) Enlarged view of the right ear showing the periotic bone, which is firmly embedded in the skull. Abbreviations: T, tympanic; P, periotic; E, exoccipital; Sq, squamosal; Pal, palatine; Max, maxilla; PF, posterior flange of the periotic.

Advancing our understanding of sound reception mechanisms in mysticetes requires a thorough exploration of both the bone and soft-tissue anatomy surrounding the ear. However, the study of soft tissues in mysticetes is particularly difficult due to the rarity of adequate specimens and the logistics of dissecting large animals, often on beaches. This study aimed to overcome these challenges in two ways. First, we focused on the minke whale (*Balaenoptera acutorostrata*), one of the smallest and most abundant mysticete species. Second, we used an integrative approach to studying the auditory anatomy through a combination of dissection, computerized tomography (CT), and MRI. Although distortion of tissues is inevitable during dissection, biomedical imaging techniques such as CT and MRI provide visualizations of internal structures *in situ*, preserving their geometries and relative positions. This is the first application of these medical imaging techniques for the study of a mysticete head and auditory system, providing an unprecedented view of the internal anatomy of these animals.

## MATERIALS AND METHODS

### Specimens

Six complete minke whale heads and one partial minke whale head were obtained from strandings in the Northeast region of the United States. The life history class/category, length, sex, carcass condition, and stranding location of each individual are given in Table 1. All complete heads were either examined fresh or frozen and kept in a −20°C freezer with no automatic thaw cycles to prevent freeze-thaw artifacts. Frozen heads were thawed before dissection. The partial head, B-acu17, was fixed in formalin.

**TABLE 1:** Minke whales used in this study

Specimen ID	Life history category	Length	Sex	Carcass condition	Stranding location
B-acu13	Subadult	389 cm	M	Code 3: Moderate Decomposition	Wellfleet, MA
B-acu15	Subadult	426 cm	M	Code 2: Fresh Dead	Sandwich, MA
B-acu17	Subadult	417 cm	F	Code 2: Fresh Dead	Brooklyn, NY
B-acu18	Subadult	430 cm	F	Code 3: Moderate Decomposition	Truro, MA
B-acu19	Subadult	465 cm	F	Code 3: Moderate Decomposition	Orleans, MA
B-acu22	Subadult	530 cm	M	Code 3: Moderate Decomposition	Vineyard Sound, MA
B-acu23	Subadult	523 cm	M	Code 3: Moderate Decomposition	Wellfleet, MA

### CT and MRI

Heads were CT scanned at 3-mm slice thickness for the whole head and rescanned at 0.1-mm slice thickness through the ear region with a Siemens Volume Zoom scanner at the Woods Hole Oceanographic Institution's Computerized Scanning and Imaging lab. In two cases where the whole head did not fit into the CT gantry (B-acu18 and B-acu19), the mandible was removed from one side of the head. Two specimens (B-acu22 and B-acu23) were too large to scan even without the mandibles. Because a reduction in tissue bulk leads to improved image quality, one of the heads (B-acu19) was trimmed to the left ear region and rescanned. The block of tissue included the left tympanoperiotic complex and surrounding bones of the skull in addition to soft tissues extending laterally to the blubber and ventrally almost to the attachment of the mandibles.

Tympanoperiotic complexes were subsequently extracted from the heads by detaching the posterior flange of the periotic bone and then cutting through the squamosal bone to free the tympanoperiotic complex. These isolated tympanoperiotic complexes were scanned by CT at 0.5-mm slice thickness. In addition, the left tympanoperiotic complex of B-acu17 was rescanned at the MRI unit at the Massachusetts Eye and Ear Infirmary in Boston, MA. Although CT uses X-ray attenuation and is superior for distinguishing between air, soft tissue, and bone, MRI uses proton density and relaxation phenomena, making it well-suited for differentiating among soft, hydrated tissues (Bushberg et al., 2002).

### Three-Dimensional Reconstructions

The internal structures of the whole minke whale head and extracted ears were reconstructed using three-dimensional visualization software AMIRA® v.5.2.2. Individual tissues were segmented using both manual selection and automated segmentation tools within AMIRA, which is more reliable than using just automated thresholding techniques (Cranford et al., 2008b). The CT scans from B-acu13 were used as the primary dataset because it was the smallest specimen, resulting in the best image quality. Data from CT scanning and dissections of all specimens were used to verify the tissue boundaries in B-acu13. A separate reconstruction was also done for the smaller section around the left ear of B-acu19.

### Dissection

Photodocumented dissections took place at the Woods Hole Oceanographic Institution's marine mammal necropsy facility and were used to verify the tissue boundaries of the three-dimensional reconstructions. The auditory region was approached from the ventral side in all specimens except for B-acu15, which was dissected from the posterior of the head, and B-acu17, which had already been dissected to expose the ear region when it was received.

From the ventral side, the mandibles were removed by cutting as close to the bone as possible. Investigation of the soft-tissue anatomy was followed by extraction of the tympanoperiotic complex, which is a technically challenging procedure in mysticetes because the fragile connections between the periotic and tympanic bones are easily broken during attempts to dislodge the tympanoperiotic complex from the skull. Once all soft tissues were removed from the area, the posterior flange was detached using an oscillating autopsy saw. The anterior flange of the periotic was freed using bone shears by incrementally chipping the thin sheet of squamosal bone lateral to the tympanic bone. Severing the soft tissue connections from inside the braincase helped to loosen the tympanoperiotic complex as well.

## RESULTS

In all minke whales examined, there was a distinct, depigmented (white) line on the epidermis projecting posteriorly from the aperture of the external auditory meatus. This marker is rarely, if ever, mentioned in the literature but would be helpful in locating the minuscule external auditory meatus. The auditory canal appeared to be continuous from its external opening to the glove finger, though winding and narrow.

**Fig 2:**
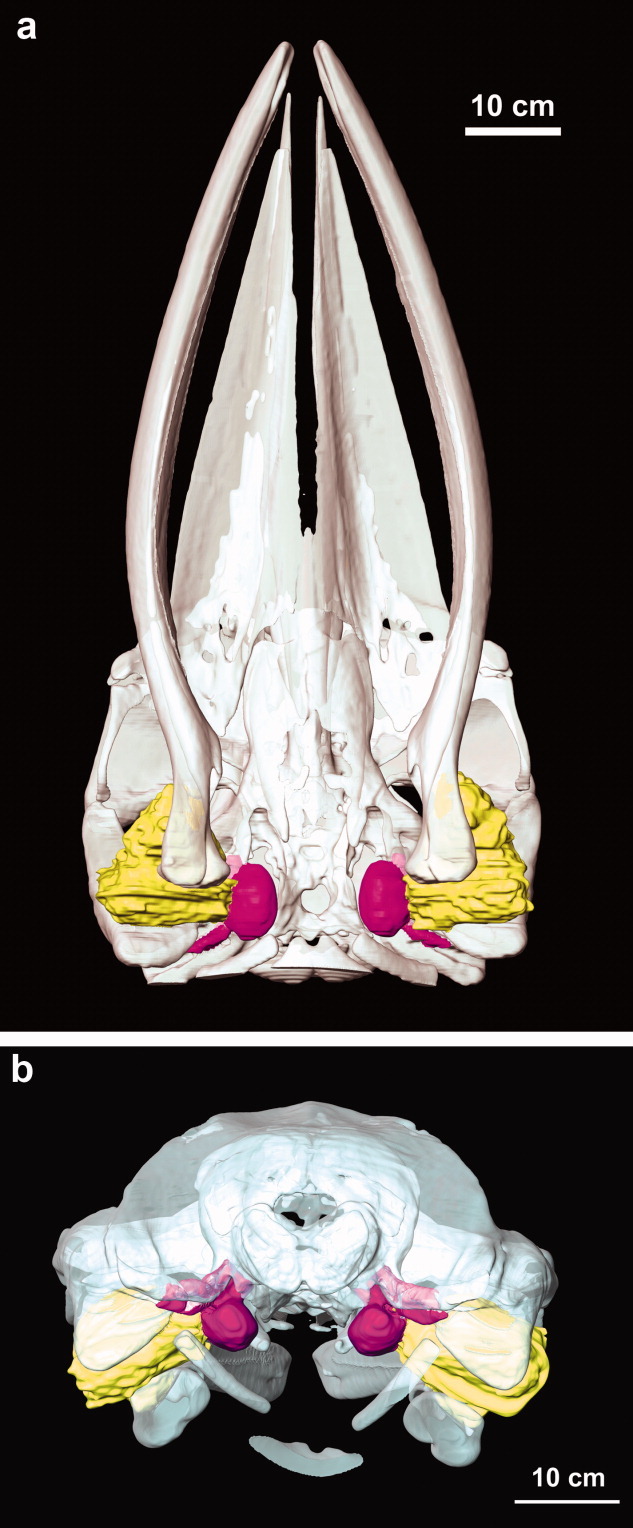
Three-dimensional reconstructions showing the contact between the ear fats and the tympano-periotic complex (ears) in the minke whale. The mandibles are still attached. (a) Ventral view. (b) Posterior view. Yellow, ear fats; purple, tympanoperiotic complex; white, other bones.

The CT images showed a large, well-formed fat body lateral, dorsal, and posterior to the mandibular ramus, ventral to the squamosal bone, and lateral to the tympanoperiotic complex. This fat body will be referred to as “ear fat” (Fig. 2). Preliminary results from lipid extractions on ear fat tissues suggest that some regions are made up of >80% lipid by wet weight (Yamato et al., 2011). The CT images and dissections indicated that the ear fat bundle became more fibrous ventrally and is integrated with the fibrous joint with the mandible. The posterior portion of the ear fat is also more fibrous, affording an attachment to the posterior margin of the squamosal bone.

**Fig 3:**
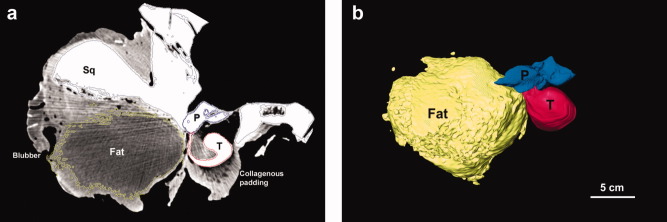
Posterior view of the partially dissected left ear region of B-acu19. (a) Axial CT image showing the ear fat extending from the blubber region to the tympanoperiotic complex (ears). Most of the blubber has been trimmed, but the remaining parts can be seen on the far left side of the image. The collagenous padding is covering the ventral portion of the tympanic bone. (b) Three-dimensional reconstruction. Yellow, ear fats; blue, periotic; red, tympanic. Abbreviations: T, tympanic; P, periotic; Sq, squamosal.

**Fig 4:**
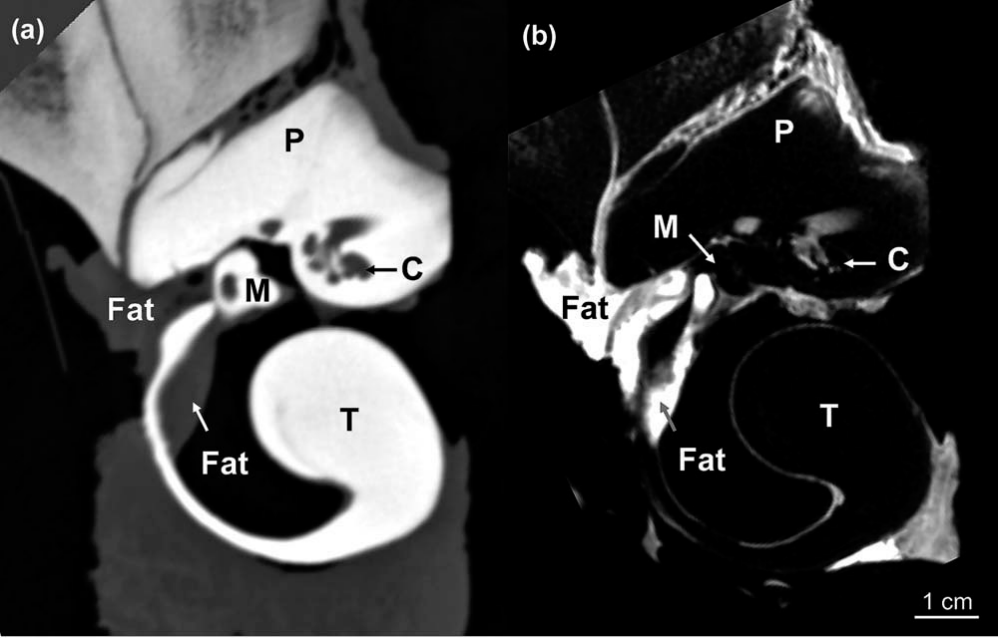
Images of the left tympanoperiotic complex of B-acu17 showing the ear fat inserting into the ears at the lateral aperture between the tympanic and periotic bones (left side of the images) and then attaching to the malleus. The smaller fat body within the tympanic bone is also shown. (a) CT and (b) MRI. Abbreviations: T, tympanic; P, periotic; M, malleus; C, cochlea.

**Fig 5:**
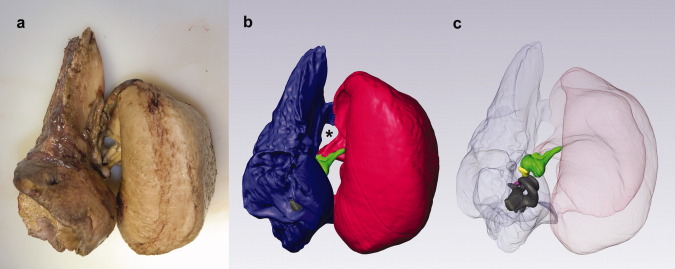
Medial view of the left tympanoperiotic complex. (a) Photograph from B-acu22. The posterior flange of the periotic has been removed to facilitate extraction. (b) Three-dimensional reconstruction for B-acu17 in approximately the same orientation as (a). The ear fat inserts into the triangular opening, which is indicated by an asterisk. (c) Same reconstruction as (b) with the tympanic and periotic bones made transparent. Blue, periotic; red, tympanic; green, malleus; yellow, incus; purple, stapes; gray, cochlea.

From the ventral perspective, the ear fat has a somewhat triangular shape with the three prominences contacting the blubber region (lateral), tympanoperiotic complex (medial), and the mandible (anterior; Fig. 2). Thus, a portion of the ear fat extends from the blubber region to the tympanoperiotic complex (Fig. 3). The anterior portion of the ear fat is well removed from the blubber layer and is adjacent to muscle. The ear fat attaches to the tympanoperiotic complex at the lateral aperture between the tympanic and periotic bones, inserting into the space that Mead and Fordyce (2009) term the “triangular opening” (Figs. 2–5). Although direct contact with the glove finger could not be determined, the ear fat is pressed against an area of the tympanoperiotic complex including the ventral portion of the glove finger. At the entry to the middle ear, the ear fat contacts the malleus (Fig. 4).

Within the middle ear space, the malleus also contacts a smaller fat pad attached to the inner wall of the tympanic bone, adjacent to the base of the glove finger (Fig. 4). The CT and MRI of the tympanoperiotic complex show these structures clearly, and they are readily visible on careful dissection. The malleus was attached to the inside of the glove finger by a strong ligamentous connection, consistent with previous reports (Lillie, 1910). Although the smaller fat pad attaches to the base of the glove finger inside the tympanoperiotic complex, neither of the fat bodies extend into the distal regions of the internal surface of the glove finger.

The tympanic bone was covered in a thick, dense, white padding composed of collagenous tissues on all sides except for the dorsal aspect (where the periotic is) and the lateral aspect, at the insertion of the ear fat into the tympanoperiotic complex (Fig. 3). The innermost layer of the padding was somewhat fatty, loosely adhering to the ventral surface of the tympanic bone. The outer portion of the padding contained irregularly dispersed cavities. The thickest portion of the padding was ∼5-cm deep.

## DISCUSSION

Sound reception in terrestrial mammals involves an air-filled outer ear. In odontocetes, which receive sound under water, the air-filled ear canal has been replaced by multiple lobes of fatty tissues leading to the tympanoperiotic complex (Norris, 1968). Two of the fat lobes are oriented anteriorly from the ears, including the inner fats filling the enlarged mandibular hiatus and the outer fats covering the lateral and ventral portions of the mandible (Ketten, 1994). These two anterior lobes are separated by the mandible, which has a thinned region termed the “pan bone” (Norris, 1968). Although Norris (1968) states that this “thin bone is transparent to the sounds used by porpoises,” the precise role of the pan bone in odontocete sound reception is still unclear (Ketten, 2000; Cranford et al., 2008a). In addition to the inner fat body and the outer fat body, a third fat lobe is located lateral to the tympanoperiotic complex and is thought to be a better sound reception pathway for lower frequency sounds (Bullock et al., 1968; Renaud and Popper, 1975; Popov and Supin, 1990; Ketten, 1994, 1997; Popov et al., 2008). All fatty lobes have well-defined connections with the tympanoperiotic complex.

The mechanism for sound reception in mysticetes is currently unknown, and no “acoustic fats” have been reported in mysticetes to date. However, our anatomical observations indicate that mysticetes also possess fat bodies associated with their ears. The contact point between the minke whale ear fat and the tympanoperiotic complex is similar to the area of contact between odontocete acoustic fats and their tympanoperiotic complex. Although the odontocete acoustic fats contact a larger surface area of the tympanoperiotic complex, the minke whale ear fats taper to insert into the “triangular opening” (Mead and Fordyce, 2009) of the tympanoperiotic complex. Inside the tympanoperiotic complex, the ear fats contact the ossicles. Laterally, the ear fat extends from the ossicles to the blubber region. Thus, the ear fats may provide a direct pathway for sound to reach the ossicles and the inner ear.

Although odontocete acoustic fats are composed of both anteriorly oriented and laterally oriented fat lobes, an exclusively lateral sound reception pathway in baleen whales is appealing. Baleen whales do not have an enlarged mandibular hiatus to house fats with any acoustic function or a thin “pan bone” region in the mandible. Balaenopterid whales like the minke whale also lunge-feed, dropping their mandibles by almost 90°. Although the ear fats would certainly be distorted during feeding, an anteriorly oriented sound reception pathway along the mandibles would be even more displaced.

The location of the ear fats somewhat overlaps with the area of the temporomandibular joint, which is currently being addressed in a separate study. Analogous to the multipurpose odontocete mandible, which is involved in both feeding and sound reception, it is possible that the mysticete ear fat is involved in other functions besides sound reception. In fact, the existence of some fatty tissue in this area of the head had been reported previously in the context of the temporomandibular joint (Hunter, 1787; Beauregard, 1882; Lambertsen et al., 1995). However, the relationship between this fatty tissue and the ears has never been explored. Interestingly, Yamada (1953) briefly noted that “similar tissue structures [as odontocetes] are seen in the impression in front of the sigmoid process” (which is between the triangular opening and the glove finger on the tympanic bone) in his study of blue (*Balaenoptera musculus*), sei (*Balaenoptera borealis*), and fin (*Balaenoptera physalus*) whales. However, he did not give a description of the tissue and it is not clear whether he is referring to the ear fat reported here. Furthermore, Yamada (1953) did not agree with a soft-tissue sound reception pathway in cetaceans and his work predated Norris's theory on odontocete sound reception. Thus, ours is the first study to describe the fat bodies located lateral to the tympanoperiotic complex as a potential sound reception pathway in mysticetes.

Similar to odontocetes, the minke whale ear canal is narrow, winding, and most likely a vestigial part of the auditory system. Although we propose the ear fats to be a primary sound reception pathway in the minke whale, it is also possible that additional mechanisms of sound reception may exist in baleen whales. For example, vibrations of the whole skull could cause differential motion between the periotic bone, which is firmly attached to the skull, and the ossicles. However, this bone conduction mechanism is less suited to produce sound localization cues compared to the proposed soft-tissue sound reception pathway. It is noteworthy that in some beaked whale species (Ziphiidae) and the sperm whale (Physeteridae), the tympanoperiotic complex also maintains a firm, osseous connection with the skull (Yamada, 1953). Yet, the primary sound reception pathways are considered to be through soft tissues for these species (Ketten and Wartzok, 1990; Ketten, 2000). Interestingly, in a preliminary study, the area of ear fat attachment in the minke whale tympanic bone (thin portion near the triangular opening) was stimulated at 40-nm amplitude with frequencies of 20 Hz–50 kHz using a piezoelectric stack to simulate incoming sound. This resulted in a movement of the stapes at the oval window, the input to the cochlea (Tubelli et al., 2012; Zosuls, personal communication).

An additional finding is that the majority of the tympanic bone is surrounded by a thick, collagenous padding except laterally, at the point of insertion of the ear fat, and dorsally, where the periotic bone is found. Odontocete tympanic bones are also partially covered by a fibrous padding, although it is much less developed than the padding in the minke whale. The same padding was described in a humpback whale (*Megaptera novaeangliae*) by Lillie (1915) as having an inner layer comprised fatty tissue and yellow elastic tissue, and an outer layer composed of spongy tissue with air cavities. Such coloration and distinct boundaries between tissue layers could not be seen in the minke whale specimens, but some cavities could be seen on the outer portion of the padding. It was unclear whether these cavities were filled with air. Yamada's (1948) description of the padding in the fin whale and the blue whale more closely match our observations. He describes the padding as a “white, thick, and hard layer of connective tissue,” which is fibrous but is loosely joined to the surface of the tympanic bone because of a fatty inner layer. Although this collagenous padding may be protecting the tympanic bone from external stresses, it may also impair sound transmission of signals from locations other than the ear fat especially if the small cavities are air-filled *in vivo*, as described by Lillie (1915).

Preliminary investigations of the fin whale and the humpback whale indicate that they have similar ear fat anatomies as the minke whale. Therefore, we hypothesize that the ear fats act as an important sound reception pathway in at least the balaenopterid family. It would be interesting to examine the soft-tissue anatomy surrounding the ears of balaenid whales, such as the North Atlantic right whale (*Eubalaena glacialis*) and the bowhead whale (*Balaena mysticetus*), which are skim feeders and have very different temporomandibular anatomies compared to the lunge-feeding balaenopterids (Eschricht and Reinhardt, 1866; Lambertsen et al., 2005).

**Fig 6:**
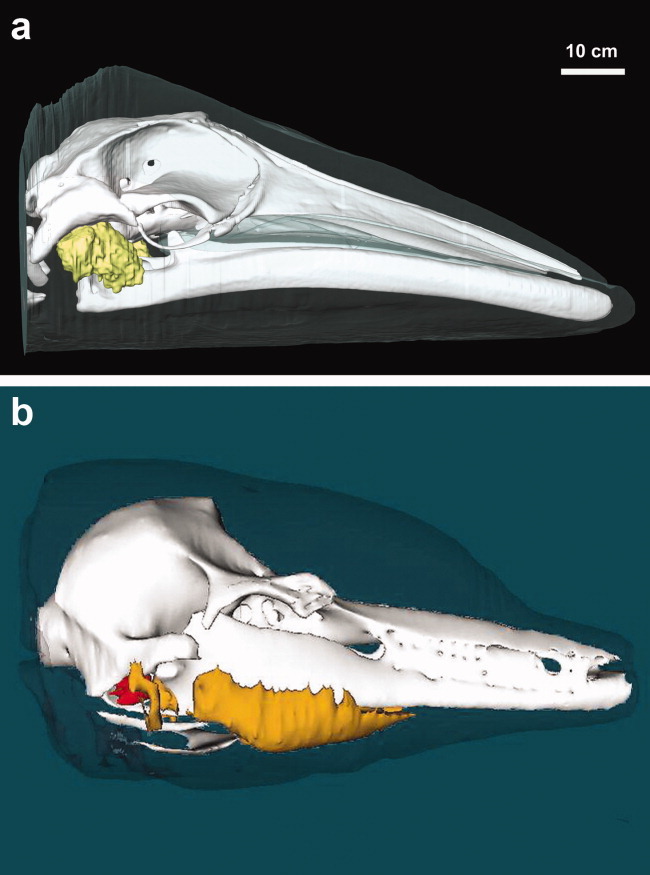
(a) Lateral view of the minke whale ear fat (yellow). (b) Lateral view of the bottlenose dolphin acoustic fats, from Ketten et al. (1999). The fatty lobe closest to the ears represents the lateral fat channel, which is more sensitive to lower frequency sounds. Orange, acoustic fats; red, tympanoperiotic complex.

Although there are many unanswered questions regarding mysticete hearing, our study suggests that fatty sound reception pathways may also exist in mysticete cetaceans. The lateral orientation of the ear fats, combined with vocalization and anatomical data indicating that mysticetes are likely to hear at low frequencies (Ketten et al., 1999; Ketten, 2000), suggest that the mysticete ear fats could be analogous to the lateral low-frequency sound reception pathway found in some odontocete species (Fig. 6). It is hypothesized that the mysticete ear fats and odontocete acoustic fats share a common evolutionary origin and developed into a more sophisticated, multilobed structure specialized for high-frequency hearing and echolocation in odontocetes. Although physiological validation studies are not yet feasible for most mysticete species, future work stemming from our anatomical study could potentially lead to a unified theory of underwater sound reception in all cetaceans.
